# Irreversible Renal Damage after Transient Renin-Angiotensin System Stimulation: Involvement of an AT_1_-Receptor Mediated Immune Response

**DOI:** 10.1371/journal.pone.0057815

**Published:** 2013-02-28

**Authors:** Bart F. J. Heijnen, Jelly Nelissen, Helma van Essen, Gregorio E. Fazzi, Jan W. Cohen Tervaert, Carine J. Peutz-Kootstra, John J. Mullins, Casper G. Schalkwijk, Ben J. A. Janssen, Harry AJ. Struijker-Boudier

**Affiliations:** 1 Department of Pharmacology, Cardiovascular Research Institute Maastricht, Maastricht University, Maastricht, The Netherlands; 2 Department of Immunology, Cardiovascular Research Institute Maastricht, Maastricht University, Maastricht, The Netherlands; 3 Department of Pathology, Cardiovascular Research Institute Maastricht, Maastricht University, Maastricht, The Netherlands; 4 Department of Internal Medicine, Cardiovascular Research Institute Maastricht, Maastricht University, Maastricht, The Netherlands; 5 Centre for Cardiovascular Science, University of Edinburgh Medical School, Edinburgh, United Kingdom; INSERM, France

## Abstract

Transient activation of the renin-angiotensin system (RAS) induces irreversible renal damage causing sustained elevation in blood pressure (BP) in Cyp1a1-Ren2 transgenic rats. In our current study we hypothesized that activation of the AT_1_-receptor (AT_1_R) leads to a T-cell response causing irreversible impairment of renal function and hypertension. Cyp1a1-Ren2 rats harbor a construct for activation of the RAS by indole-3-carbinol (I3C). Rats were fed a I3C diet between 4–8 weeks of age to induce hypertension. Next, I3C was withdrawn and rats were followed-up for another 12 weeks. Additional groups received losartan (20 mg/kg/day) or hydralazine (100 mg/kg/day) treatment between 4–8 weeks. Rats were placed for 24h in metabolic cages before determining BP at week 8, 12 and 20. At these ages, subsets of animals were sacrificed and the presence of kidney T-cell subpopulations was investigated by immunohistochemistry and molecular marker analysis. The development of sustained hypertension was completely prevented by losartan, whereas hydralazine only caused a partial decrease in BP. Markers of renal damage: KIM-1 and osteopontin were highly expressed in urine and kidney samples of I3C-treated rats, even until 20 weeks of age. Additionally, renal expression of regulatory-T cells (Tregs) was highly increased in I3C-treated rats, whereas the expression of T-helper 1 (Th1) cells demonstrated a strong decrease. Losartan prevented these effects completely, whereas hydralazine was unable to affect these changes. In young Cyp1a1-Ren2 rats AT_1_R activation leads to induction of an immune response, causing a shift from Th1-cells to Tregs, contributing to the development of irreversible renal damage and hypertension.

## Introduction

One of the characteristics during structural remodeling of target organs in hypertension is the presence of a distinct inflammatory response [1]. Inflammation contributes to structural changes by activation and/or inhibition of growth factors and proteases and might be the predisposing factor for hypertension induced injury [2]. The link between hypertension and inflammation has been widely studied and several mechanisms and pathways are proposed [3], [4]. Angiotensin (Ang) II induces renal damage, both in the presence and absence of hypertension [2]. In the past it has been shown that Ang II when infused in animals at a dose below the threshold of its vasoconstrictor effect (subpressor dose), BP rises slowly and progressively over time [5], [6]. Thus far, the exact cause of this so-called late low-pressor response is still unclear.

Over the last two decades, Ang II has been recognized as an important inducer of inflammation [2], [7]. Binding of Ang II to the angiotensin II type 1 receptor (AT_1_R) activates nuclear factor-kappa B (NFκB) via upregulation of several proinflammatory genes, thereby contributing to a local inflammatory response [2]. Furthermore, Ang II causes infiltration of inflammatory cells, including macrophages and T-cells, in glomeruli and interstitium [7]–[9]. The importance of a T-cell response in the pathophysiology of hypertension induced injury has been established previously [1], [10], [11]. Mice lacking T-cells develop less severe hypertension than their wild type counterparts [7]. Some recent studies demonstrate that a particular subpopulation of T cells, the regulatory T cell (Treg), is able to prevent Ang II and aldosterone-induced hypertension and injury [12], [13]. However, the exact mechanisms along which the different subtypes of T-cells contribute to the development and progression of hypertension-induced renal injury is incompletely understood. Nataraj and co-workers presented one of the first studies that demonstrate a direct action of Ang II on T lymphocytes via the AT_1_R [14]. The role of the AT_1_R in this process remains controversial. Whereas numerous studies provide data that activation of the AT_1_R is unfavorable, a recent study by Zhang et al [15] shows that the AT_1_R expressed on T lymphocytes can limit target organ damage in hypertension. Kidney injury molecule-1 (KIM-1) and osteopontin (OPN) are recognized as regulators of T-cells and not surprisingly they are also known as important renal injury markers [16], [17]. In patients AT_1_R blockade reduces renal damage and is associated with less excretion of KIM-1 and OPN [18]. Therefore we hypothesize that these markers might play an important role via immune activation in angiotensin-induced renal pathogenesis.

In a recent study, we demonstrated that transient stimulation of the renin-angiotensin system leads to sustained high BP in young Cyp1a1-Ren2 transgenic rats due to the induction of irreversible renal damage [19]. These rats harbour a genetic construct under control of a Cyp1a1 promoter for the production of mouse renin 2 (mRen2) [20]. This promoter can be activated by adding indole-3-carbinol (I3C) to the diet eventually leading to RAS-activation via increased circulating prorenin- and renin-levels [21], [22]. In the current study we hypothesized that the induction of irreversible renal damage in this model is mainly aggravated by an AT_1_R activated immune response. For this reason we compared hypertensive losartan- with hydralazine-treated Cyp1a1-Ren2 rats.

## Animals, Materials and Methods

### Ethics Statement

All animal experiments were performed in accordance with guidelines issued in the “Guide for the Care and Use of Laboratory Animals” (2010) and were approved by the institutional Animal Care and Use Committee of Maastricht University.

### Animals and Experimental Procedures

Transient malignant hypertension was induced in transgenic young Cyp1a1-Ren2 rats by feeding the animals a diet containing 0.3% I3C (Sigma-Aldrich, St. Louis, Missouri, USA) between 4 and 8 weeks of age. As shown previously, BP remains elevated in these rats up to 12 weeks after withdrawal of I3C and is associated with irreversible renal damage accompanied by a moderate inflammatory response [19].

First, we tested whether the elevation in BP and the development of renal damage due to 4 weeks of RAS-stimulation in Cyp1a1-Ren2 rats was prevented by AT_1_R-blockade via losartan-treatment between 4–8 weeks of age. Losartan (MSD, Oss, the Netherlands) was dissolved in PBS (GIBCO, Life technologies, Carlsbad, CA, USA) and administered at a dose of 20 mg kg^−1^ day^−1^ via subcutaneously (s.c.) implanted osmotic minipumps (MODEL 2004 Alzet, Durect Corporation, Cupertino, CA, USA). The osmotic minipumps were primed 24 hours prior to implantation under isoflurane anesthesia (1–4% Forane, Abbott House, Berkshire, UK) and buprenorphine analgesia (0.03 mg kg^−1 ^s.c. TEMGESIC).The dose of losartan was based on previous studies by our group [23]. To demonstrate the selectivity of AT_1_R activation, an additional group of RAS-stimulated rats received hydralazine (Sigma-Aldrich) treatment between 4–8 weeks of age. Hydralazine was administered via drinking water at a dose of 100 mg kg^−1^ day^−1^. To check and adapt treatment for proper dosing, rats were weighed and drinking water with the appropriate dilution of hydralazine was freshly prepared every two days. In addition, rats received s.c. an osmotic minipump containing hydralazine at the highest possible dose (3 mg kg^−1^ day^−1^) serving as a sham procedure and to boost antihypertensive treatment with this agent. The effects of the hydralazine treatment were compared to the age-matched control, I3C-treated and I3C+losartan-treated group. At 8 weeks of age animals were placed in metabolic cages to collect 24 hour urine samples for the determination of albuminuria and the excretion of renal injury markers. Additionally, at that age direct arterial blood pressure was determined in the conscious state. For this purpose, a polyethylene catheter was inserted into the right femoral artery and shifted into the aorta under isoflurane anesthesia and buprenorphine analgesia for more than 48 hours before measuring mean arterial pressure (MAP).For each animal MAP was recorded for approximately 1 hour. Further, renal vascular resistance (RVR) was measured under pentobarbital anesthesia (50 mg kg^−1^). A transit time flow probe (VB series 0.5 nm, Transonic Systems Inc., Ithaca, New York, USA) was placed around the renal artery to measure renal blood flow (RBF). After stabilization of hemodynamics (>20 min), RBF and MAP were recorded simultaneously. Renal vascular resistance (RVR) was calculated as MAP divided by RBF and corrected for kidney weight. Afterwards, rats were sacrificed by exsanguination via the abdominal aorta and the kidneys were weighed and harvested for further molecular and immuno-histochemical analysis.

**Figure 1:**
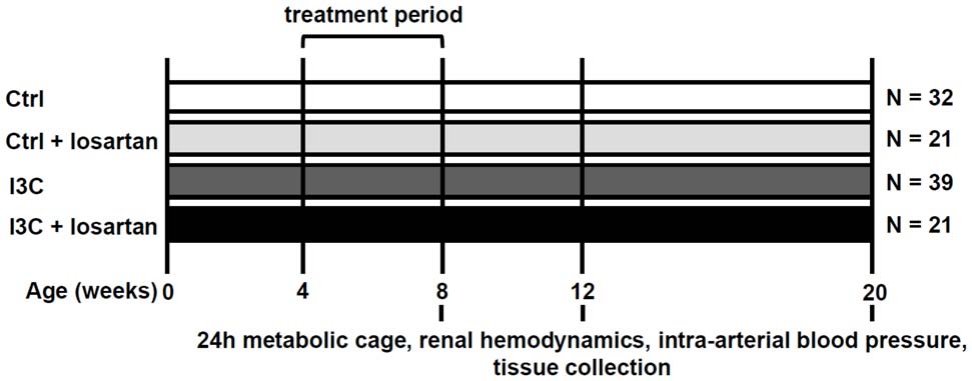
Experimental setup. Cyp1a1-Ren2 rats were treated from 4–8 weeks of age. The I3C-treated rats received 0.3% I3C via the diet, whereas control rats received standard rat chow. Losartan was administered via s.c. implanted minipumps at a dose of 20 mg kg^−1^ day^−1^. Losartan and I3C-treatment were stopped at 8 weeks of age and rats were followed-up until 20 weeks of age, i.e. 12 weeks after stopping the treatment-regiments. *Ctrl, Control; I3C, Indole-3-Carbinol.*

In a second set of experiments we further evaluated the underlying immune response and tested whether the 4 weeks of AT_1_R-blockade, during the 4 weeks I3C-treatment with high circulating (pro-)renin and Ang II levels, was able to completely prevent the development of hypertension and renal damage over time. For this purpose we included 4 groups: control, control+losartan, I3C-treated and I3C-treated+losartan, and followed them up until 20 weeks of age. At 8 weeks of age the osmotic minipumps were carefully removed under isoflurane anesthesia and buprenorphine analgesia. At 8, 12 and 20 weeks of age animals were placed in metabolic cages, direct arterial blood pressure and RVR were determined and the rats were sacrificed and the organs were processed for further analysis. Figure 1 illustrates the experimental setup of this in vivo experiment.

### Urinary Markers of Renal Injury

Albumin was measured in 24h urine samples using the Beckman/Coulter Synchron LX 20 (Beckman Coulter, Inc., Brea, California, USA). In addition, rat kidney injury panel 1 was purchased from mesoscale diagnostics (MSD; K15162C-1, Meso Scale Discovery, Gaithersburg, MD, USA). This assay detects lipocalin-2 (NGAL), osteopontin, and kidney-injury-molecule (KIM-1) in a sandwich immunoassay and uses a competitive assay format to detect albumin. All reagents were provided with the MSD kit. Each 96-well plate had 4-carbon electrodes in the bottom of each well, each pre-coated with one of the 4-anti-kidney injury markers antibodies of interest. The assay was performed according to the manufacturers’ protocol [24]. Finally, albumin and the renal injury markers were corrected for urinary creatinine, determined on the Beckman/Coulter Synchron LX 20.

### Gene Expression Analysis

Total RNA was isolated from a cross-section of the right kidney by the TRIZOL-method (Invitrogen, Carlsbad, California, USA), as previously described [19]. The purity and concentration of the RNA was measured by means of the 260/280 ratio, and cDNA synthesis was performed using 1000 ng RNA according to the manufacturers’ protocol (iScript cDNA synthesis kit, Bio-Rad Laboratories Inc., Hercules, California, USA). Gene expression was analyzed by quantitative PCR on a Bio-Rad CFX96 system (Bio-Rad Laboratories Inc) using the iQ SYBR-green supermix (Biorad Laboratories Inc). All primers applied are presented in Table 1. Results were normalized to the housekeeping gene cyclophilin A and relative changes in expression levels were subsequently calculated using the Bio-Rad CFX manager software (Version 2.0, Biorad Laboratories Inc). Relative gene expression in the control rats was normalized for each time-point to compare them with the age-matched rats from the other three groups.

**Table 1:** Gene-specific primer sequences used for quantitative real-time PCR.

Gene	Forward-primer 5′-3′	Reversed-primer 5′-3′
*CD3*	GCAACACCAGCATCAGGCAT	CCCAAAGCCAGGAGCAGAGT
*CD4*	AGGACAGTGGCATCTGGAAC	TGGGGTATCTGAAGGGTGAG
*CD8*	CCGGTCTGCCCATCATATAG	GGGCAGTTCTCTTGTCTTGG
*Cyclophilin A*	TTCCTCCTTTCACAGAATTATTCCA	CCACCAGTGCCATTATGG
*FoxP3*	CCCAGGAAAGACAGCAACCTT	CTGCTTGGCAGTGCTTGAGAA
*GATA3*	TTCCTGTGCGAACTGTCAGACCA	CCTTTTTGCACTTTTTCGATTTGCTA
*IL17*	ATCAGGACGCGCAAACATG	TGATCGCTGCTGCCTTCAC
*KIM-1*	CGGTGCCTGTGAGTAAATAGAT	CTGGCCATGACACAAATAAGAC
*Nephrin*	CGTGCTAAAGGCGAGTTCCA	GGAGAGGATTACTTTAGGAGACACAAG
*NGAL*	GACTCAACTCAGAACTTGATCCCT	AGCTCTGTATCTGAGGGTAGCTGT
*OPN*	TGAGACTGGCAGTGGTTTGC	CCACTTTCACCGGGAGACA
*PAI-1*	GAGCCAGATTCATCATCAACG	CTGCAATGAACATGCTGAGG
*T-bet*	TCCACCCAGACTCCCCCAACA	GGCTCACCGTCATTCACCTCCA
*TGFβ*	GCACCATCCATGACATGAAC	GCTGAAGCAGTAGTTGGTATC

FoxP3, forkhead box P3; GATA3, GATA binding protein 3; IL17, Interleukin 17; KIM-1, kidney injury molecule 1; NGAL, neutrophil gelatinase associated lipocalin; OPN, osteopontin; PAI-1, plasminogen activator inhibitor-1; T-bet, T-box expressed on T-cells; TGFβ, transforming growth factor β.

### Histochemisty and Immunostaining

#### PAS-D

The right kidney of each animal was excised, rinsed in PBS (Invitrogen) and fixed in 4% formalin for 24 hours before embedding in paraffin. Central cross-sections of 4 µm of the whole kidney including medulla and cortex were deparaffinized and stained for PAS-D. Briefly, the slides where incubated with diastase (Merck; 1 g per 330 ml de-mineralized water) for 90 minutes. Next, slides were washed with de-mineralized water and incubated in periodic acid (Merck) for 10 minutes, followed by a second washing step with de-mineralized water. Then the slides were subjected to 20 min incubation with Schiff reagent (Merck) and washed with tap water. All slides were counterstained with haematoxylin and rehydrated in increasing concentrations of ethanol.

All sections were semi-quantitatively scored by a nephropathologist in a blinded manner as described previously [19]. Briefly, renal scarring of all glomeruli was scored on an arbitrary scale from 0 to 4. Grade 0 indicated no glomerulosclerosis, grade 1<25% of sclerosis, grade 2 25–50% of sclerosis, grade 3 50–75% of sclerosis and grade 4>75% of sclerosis per glomerulus. Hereafter, the individual glomerulosclerosis index (GSI) was calculated for each rat. Furthermore, 10 images of each kidney section (100× magnification) were analyzed for tubular atrophy, interstitial fibrosis and total renal inflammation according to the Banff classification. Each parameter was graded a score of 0 to 3, where 0 meant no changes in pathology, grade 1<25% of change, grade 2 a 25–50% difference and grade 3>50% of affected tissue. From these data the tubulointerstitial score (TIS) was calculated.

#### WT-1

To analyze the glomerular filtration barrier and gain more insight in the glomerular structural alterations a Wilms’ tumor 1 (WT-1) staining was performed on paraffin embedded tissue. First, slides were subjected to ‘Target Retrieval Solution’ (DAKO Denmark A/S, Glostrup, Denmark) at 96°C for 10 minutes. Next, peroxidase activity was blocked by Envision flex peroxidase block (DAKO) for 5 minutes. Hereafter, the tissues were put on WT-1 ready-to-use (DAKO) for 20 minutes, Envision Flex HRP (DAKO) for 20 minutes and Diaminobenzidine (DAB) (DAKO) for 10 minutes. This final step was blocked by the addition of tap water. Finally, the tissues were counterstained with haematoxylin and dehydrated in increasing concentrations of ethanol. Analysis of WT-1 was done by light microscopy at a magnification of 200×. For each subject approximately 20 randomly selected glomeruli were analyzed throughout the entire cortex. WT-1 positive cells were counted and corrected for glomerular diameter as determined by a computerized morphometric system (Leica Qwin 3.1). The average data of all glomeruli per subject were then used in the analysis.

#### CD3

A CD3 staining was performed to determine T-cell infiltration in the kidney. First, slides were incubated for 30 minutes in 0.3% peroxide in PBS to block endogenous peroxidase activity. Next, antigen retrieval was performed by boiling the slides in a Tris EDTA buffer (1.2 g/L Tris and 0.37 g/L EDTA, pH 9) for 10 minutes. Primary antibody (mouse-α-human CD3 (DAKO), 1∶200 in PBS with 1% BSA) was incubated overnight at 4°C. Hereafter, slides were incubated for 30 minutes with secondary antibody (rabbit-α-mouse peroxydase conjugated, 1∶1000 in PBS, DAKO). Subsequently, Envision anti-rabbit incubation for 30 minutes and DAB (DAKO) was used to obtain staining of the T-cells. Finally, the tissues were counterstained with haematoxylin and dehydrated in increasing concentrations of ethanol. Analysis of CD3 was done by light microscopy at a magnification of 200x by means of 10 photos per subject throughout the entire cortex. Brown staining was measured as percentage of total tissue area using a computerized morphometric system (Leica Qwin 3.1).

### Statistical Analysis

Data are expressed as mean ± S.E.M. and were analyzed by one-way or 2-way ANOVA with post-hoc Bonferroni correction when appropriate, followed by unpaired Student’s *t* test. Differences were considered statistically significant if p-values <0.05 (* p<0.05, † p<0.01 and ‡ p<0.001).

## Results

### Effects of Anti-hypertensive Treatments

#### Hemodynamics

**Figure 2:**
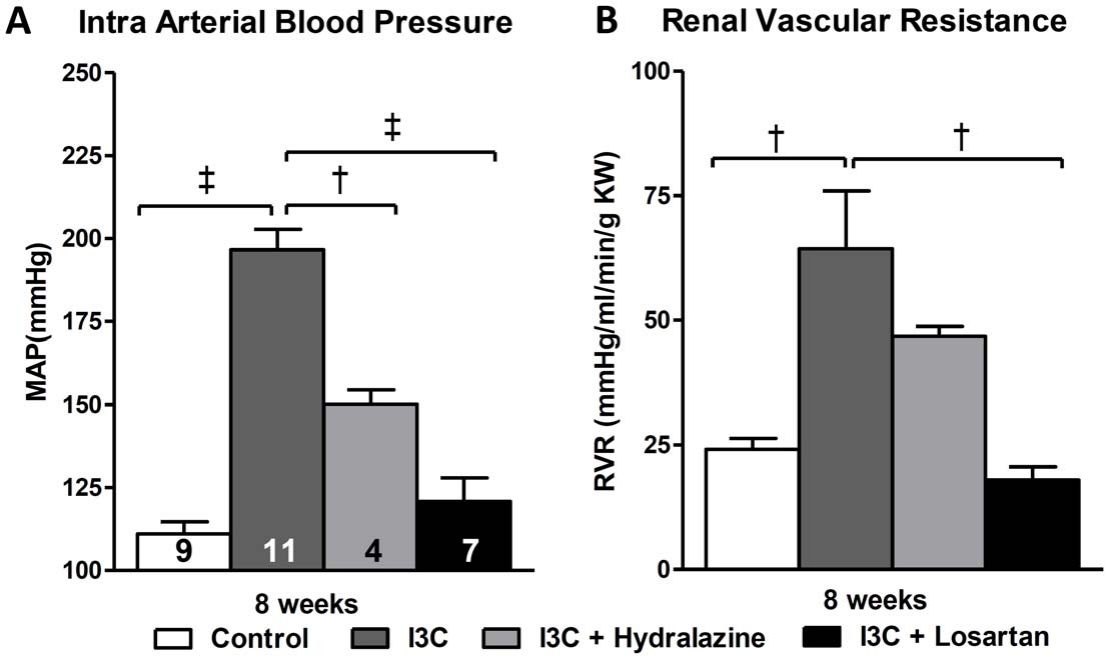
Effects of anti-hypertensive treatments on hemodynamics. At 8 weeks of age mean arterial blood pressure (MAP) (A) and renal vascular resistance (RVR) (B) were significantly elevated in the I3C-treated group after renin-angiotensin system (RAS)- stimulation. Whereas losartan-treatment was able to prevent the rise in MAP and RVR completely, hydralazine was also able to reduce them, yet MAP and RVR remained significantly elevated. The n for each group is presented in the bars of figure A. *I3C, Indole-3-Carbinol; KW, kidney weight; MAP, mean arterial pressure.*

Four weeks of RAS activation in the I3C-treated rats led to a significant increase in mean arterial pressure (MAP). Losartan-treatment between 4–8 weeks of age was able to block the rise in MAP. In addition, the RAS-independent lowering of blood pressure by hydralazine demonstrated a significant, yet only a partial reduction in MAP. RVR was also partially decreased. (Fig. 2A+B).

#### Markers of renal damage

**Figure 3:**
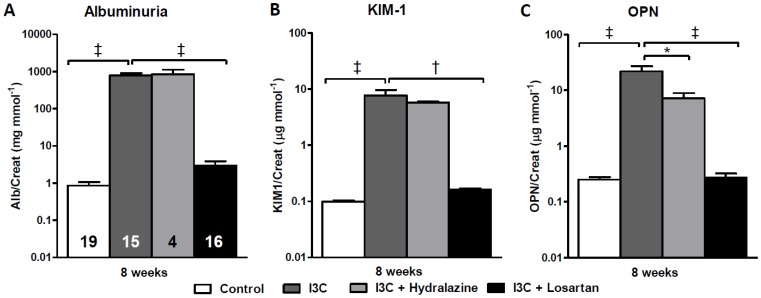
Effects of anti-hypertensive treatments on urinary excretion of renal injury markers. Whereas losartan completely prevented the development of albuminuria, hydralazine treatment was unable to induce any improvement (A). Furthermore, urinary excretion of KIM-1 and OPN was fully inhibited by AT_1_-receptor blockade. Hydralazine only induced a slight decrease of these markers, yet, they remained significantly elevated (B+C). The n for each group is presented in the bars of figure A. *I3C, Indole-3-Carbinol; KIM-1, kidney injury molecule-1; OPN, osteopontin.*

**Figure 4:**
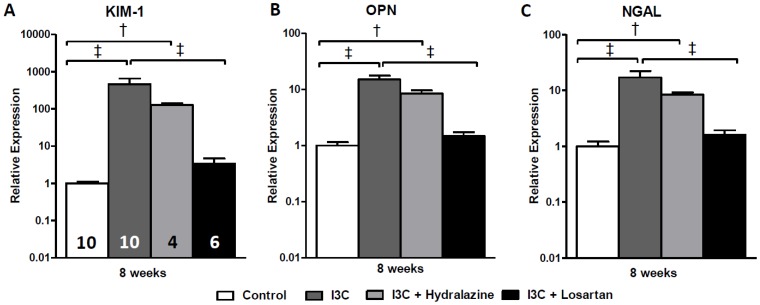
Effects of anti-hypertensive treatments on gene expression of renal injury markers. Hydralazine treated rats expressed elevated levels of the renal injury markers KIM-1, OPN and NGAL. When compared with the untreated RAS-stimulated rats, gene expression levels demonstrated a decrease yet remained significantly elevated (A-C). This was in sharp contrast with losartan which caused a normalization of all parameters. PAI-1 expression was unaltered whereas nephrin was significantly decreased by hydralazine when compared with untreated RAS-stimulated rats (D+E). The n for each group is presented in the bars of figure A. *I3C, Indole-3-Carbinol; KIM-1, Kidney injury molecule-1; NGAL, neutrophil gelatinase associated lipocalin; PAI-1, plasminogen activator inhibitor-1.*

**Figure 5:**
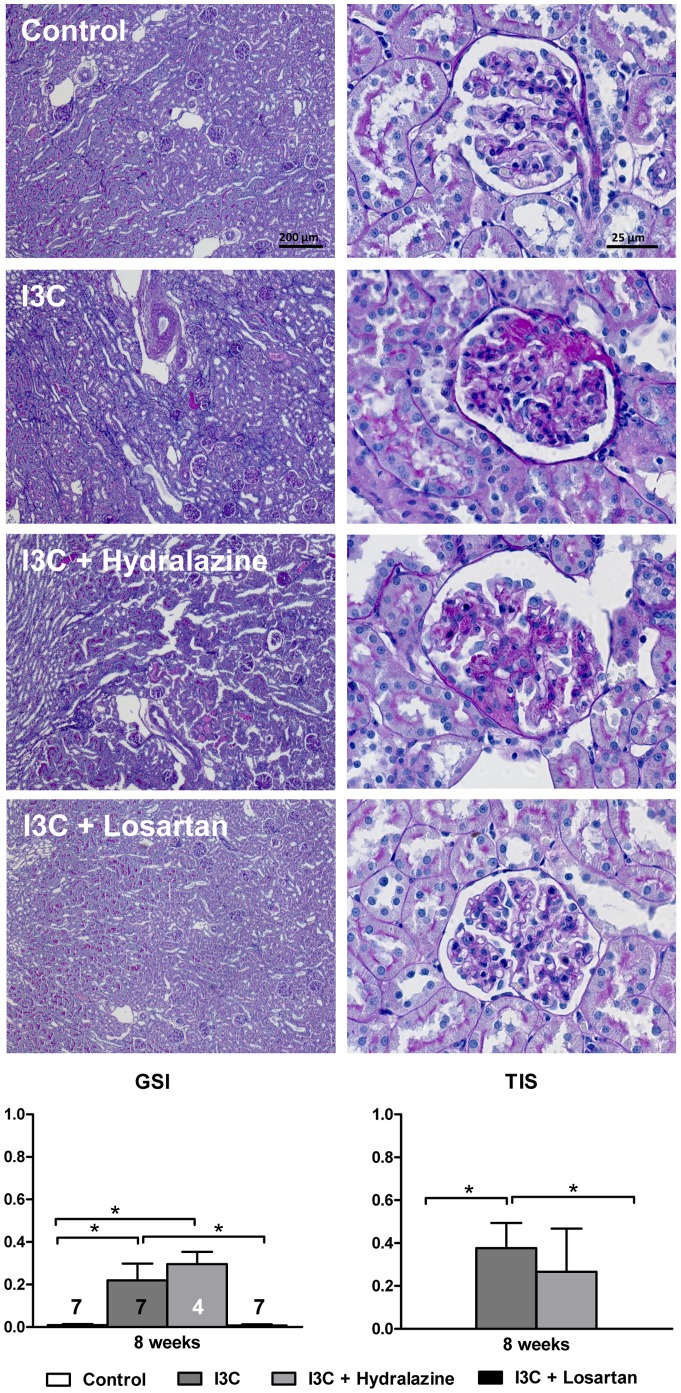
Effects of anti-hypertensive treatments on renal injury. Four weeks of RAS-stimulation caused a significant increase in renal pathology, which is reflected by the GSI and TIS. AT_1_R-blockade completely prevented this, whereas hydralazine had no effect. The n for each group is presented in the bars of the GSI. *I3C, Indole-3-Carbinol; GSI, glomerulosclerosis index; TIS, tubulointerstitial score.*

RAS-stimulated rats developed albuminuria (Fig. 3A). Furthermore, the excretion of urinary KIM-1 and OPN was significantly increased (Fig. 3B+C) and followed a similar pattern as renal gene expression (Fig. 4). Losartan-treatment fully prevented the increase in expression of these renal injury markers and inhibited the excretion of them as well as of albumin. In contrast, in hydralazine treated-rats renal expression and urinary excretion of KIM-1, NGAL and albuminuria were in the same range as non-treated RAS-stimulated rats, whereas excretion of OPN was slightly decreased. These data are supported by analysis of PAS-D stained kidney slides and determination of renal pathology by means of GSI and TIS (Fig. 5). In short, whereas losartan completely prevents the development of an increased GSI and TIS, hydralazine is unable to improve renal pathology.

### Long-term Effects with and without AT_1_R-blockade

#### Hemodynamics

**Figure 6:**
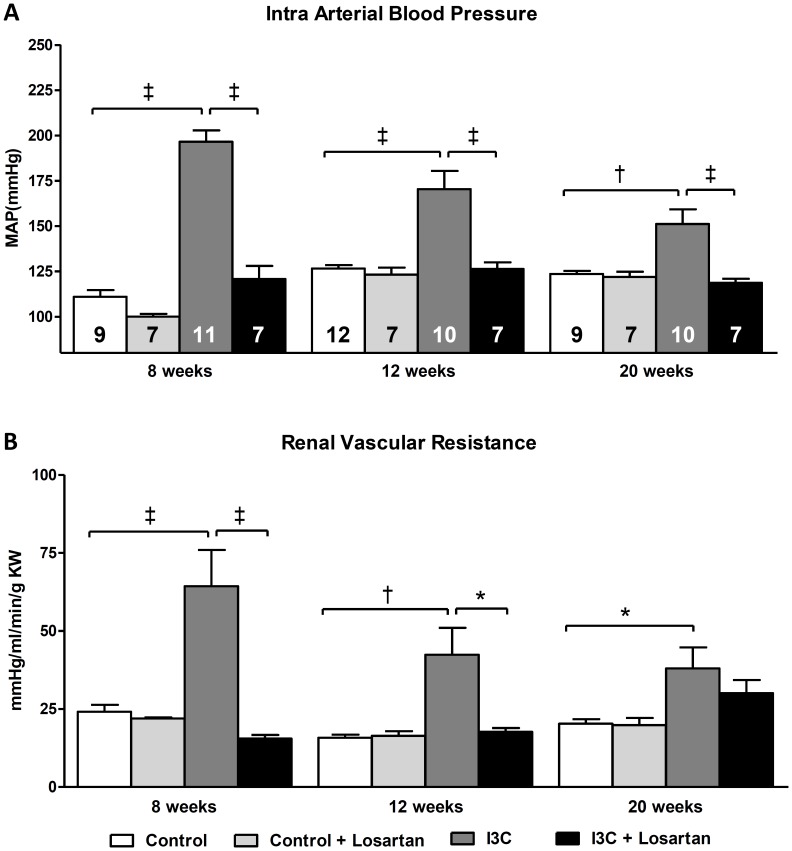
Long-term effects of AT_1_-receptor blockade on hemodynamics. Mean arterial blood pressure (MAP) (A) and renal vascular resistance (RVR) (B) were significantly elevated in the I3C-treated group after renin-angiotensin system (RAS)- stimulation between 4–8 weeks of age. Losartan-treatment was able to prevent this completely. After stopping RAS-stimulation (after week 8) MAP and RVR decreased but remained significantly elevated, even until 20 weeks of age. Losartan treatment was also able to prevent the development of this sustained high blood pressure. The n for each group is presented in the bars of figure A. *I3C, Indole-3-Carbinol; KW, kidney weight; MAP, mean arterial pressure.*

After withdrawal of I3C from the diet, MAP decreased in the I3C-treated rats but remained highly elevated at 12 weeks of age. Even at 20 weeks of age, i.e. 12 weeks after stopping RAS-stimulation, MAP remained significantly higher when compared with the control rats. RVR demonstrated the same pattern. Four weeks of AT_1_R-blockade was able to completely prevent these effects. (Fig. 6A+B).

#### Markers of renal damage

**Figure 7:**
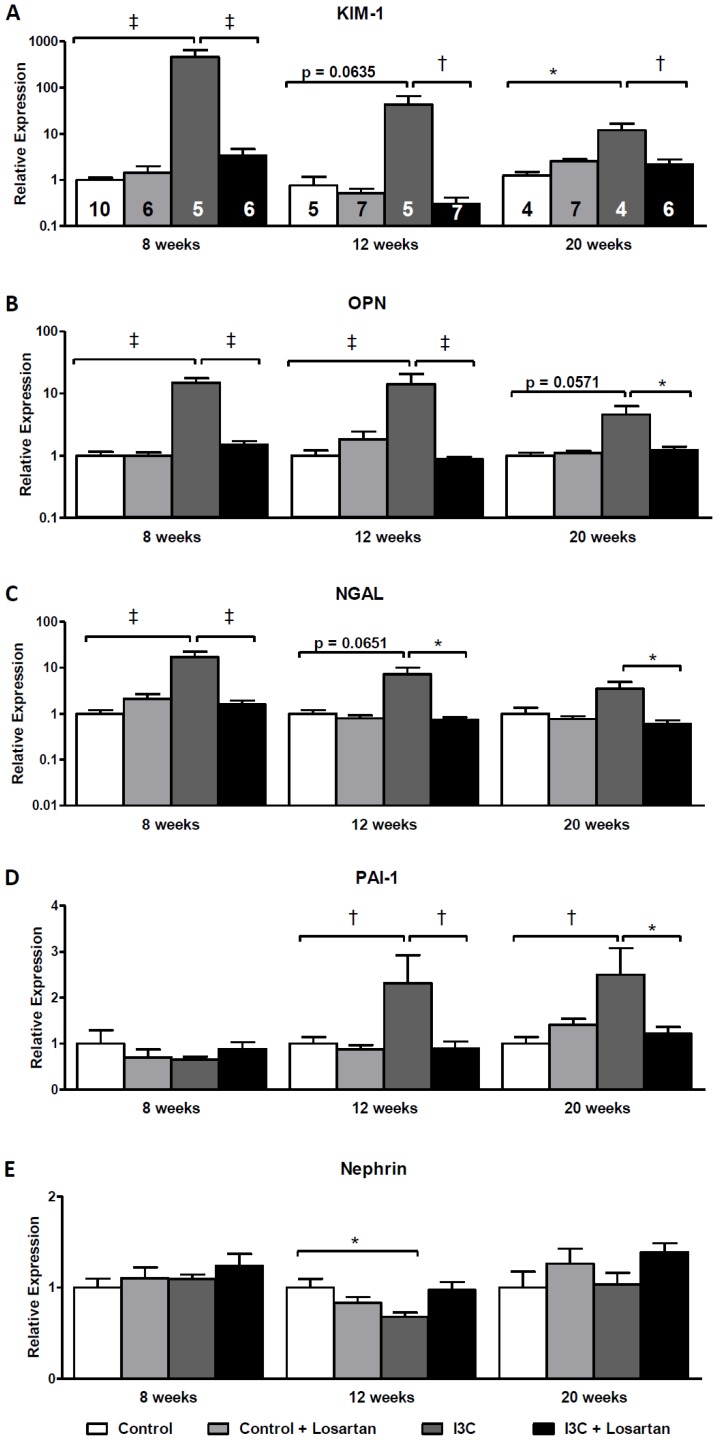
Long-term effects of of AT_1_-receptor blockade on gene expression of renal injury markers. Gene expression of the renal injury markers NGAL, KIM-1 and OPN was highly elevated after 4 weeks of RAS-stimulation (A–C). Stopping RAS-stimulation led to a decrease in expression, nevertheless, the levels remained significantly elevated even until 20 weeks of age. At first, PAI-1 expression remained at control levels and was increased at 12 and 20 weeks of age in the RAS-stimulated rats (D). Finally, the expression of nephrin demonstrated little to no differences (E). Only a small decrease in expression was observed at 12 weeks of age in the RAS-stimulated rats. Losartan-treatment was able to prevent all of the effects on the renal injury markers. In adult rats which do not develop sustained hypertension after 4 weeks of RAS-stimulation a normalization of all renal injury markers occurs within 4 weeks after stopping the stimulation (data not shown). The n for each group is presented in the bars of figure A. *I3C, Indole-3-Carbinol; KIM-1, kidney injury molecule-1; NGAL, neutrophil gelatinase associated lipocalin; OPN, osteopontin; PAI-1, plasminogen activator inhibitor-1.*

**Figure 8:**
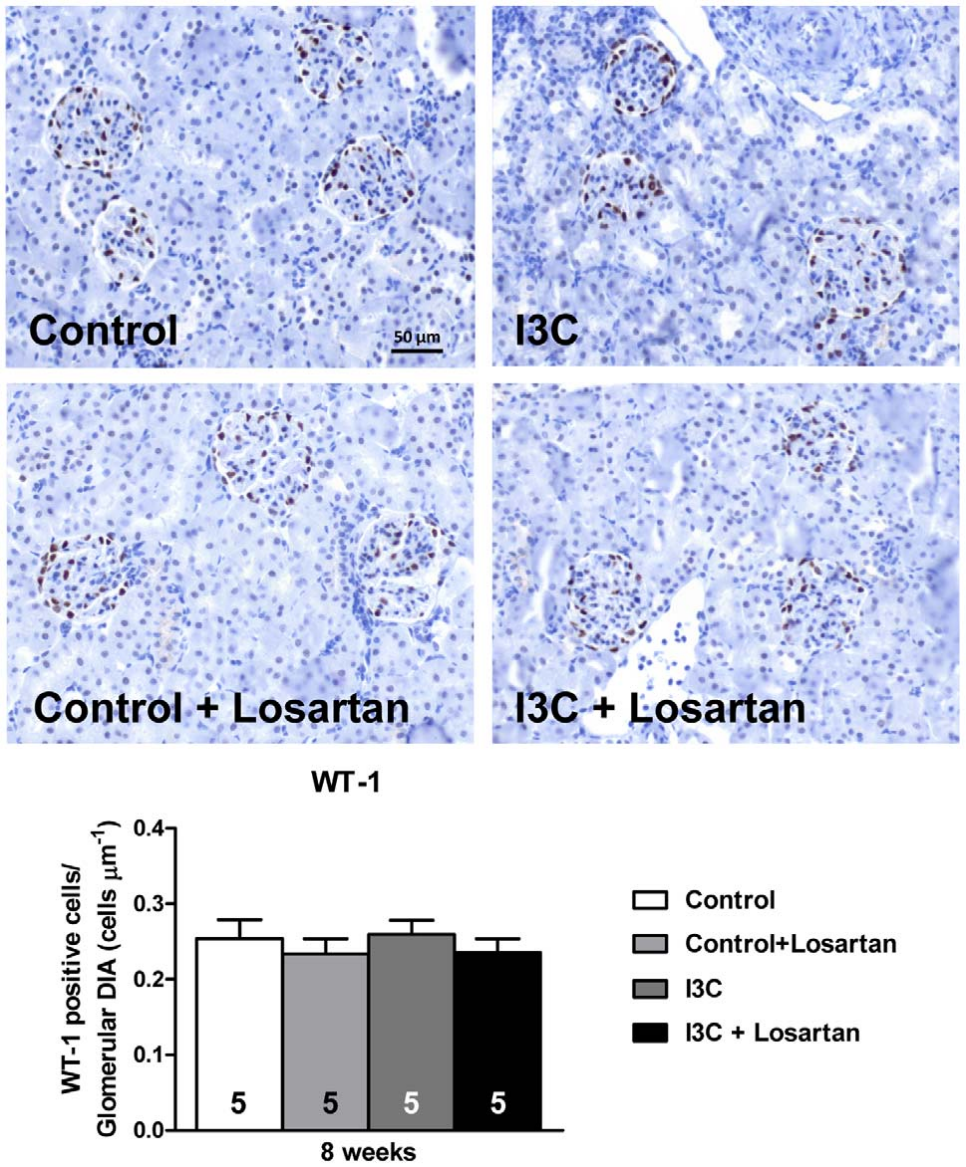
Expression of glomerular WT-1. RAS-stimulated rats, with or without AT_1_-receptor blockade, did not demonstrate differences in WT-1 positive cells when compared with normotensive controls. The n for each group is presented in the bars of the figure. *DIA, diameter; I3C, Indole-3-Carbinol; WT-1, Wilms’ tumor 1.*

Stopping RAS-stimulation led to a decrease in expression of the renal injury markers KIM-1, OPN and NGAL. Nevertheless, the levels remained significantly elevated even until 20 weeks of age (Fig. 7A–C). Whereas the increase of these markers was present immediately at 8 weeks of age, PAI-1 expression remained at control levels at first. Later on, PAI-1 demonstrated increased expression levels in the RAS-stimulated rats at 12 and 20 weeks of age (Fig. 7D). Finally, the expression of nephrin demonstrated little to no differences (Fig. 7E). Only a small decrease in expression was observed at 12 weeks of age in the RAS-stimulated rats. Further evaluation of the glomerular filtration barrier was done by immunohistochemical analysis of WT-1 expression at 8 weeks of age, i.e. when albuminuria was most prominent. Again no differences were observed (Fig. 8).

**Figure 9:**
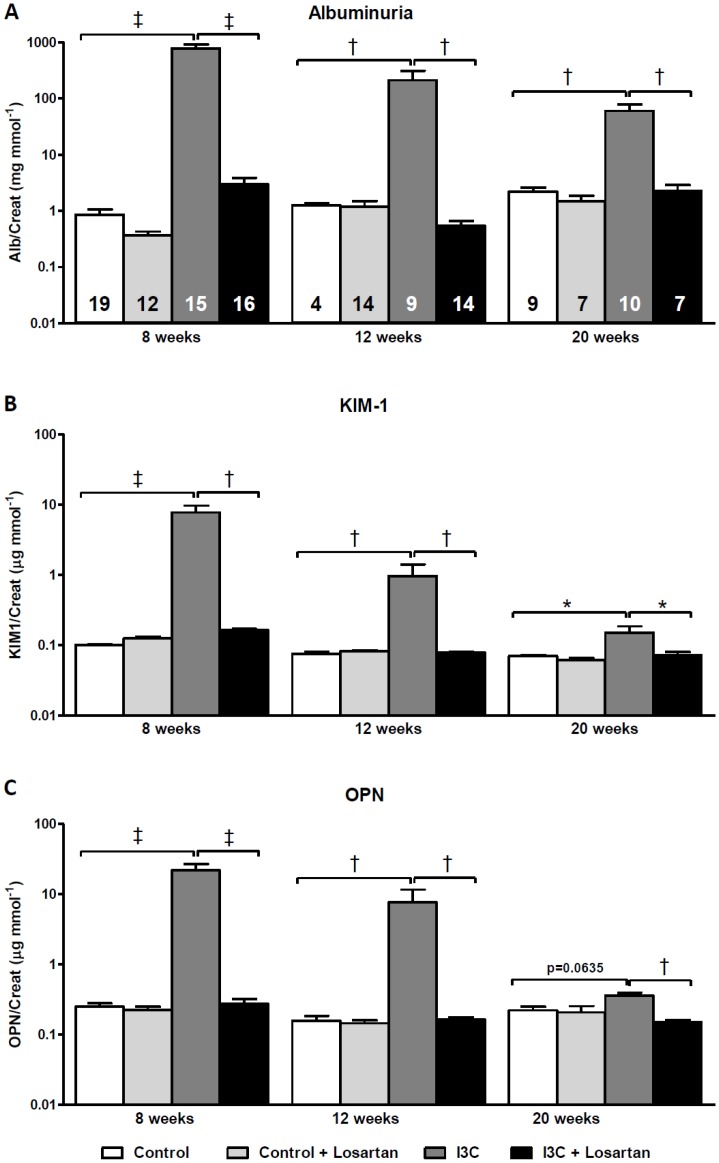
Long-term effects of AT_1_-receptor blockade on urinary excretion of renal injury markers. RAS-stimulated rats developed sustained albuminuria (A). The excretion of urinary KIM-1 and OPN demonstrated the same pattern (B+C). The excretion of all renal injury markers was completely prevented by losartan. The n for each group is presented in the bars of figure A. *I3C, Indole-3-Carbinol; KIM-1, kidney injury molecule-1; OPN, osteopontin.*

**Figure 10:**
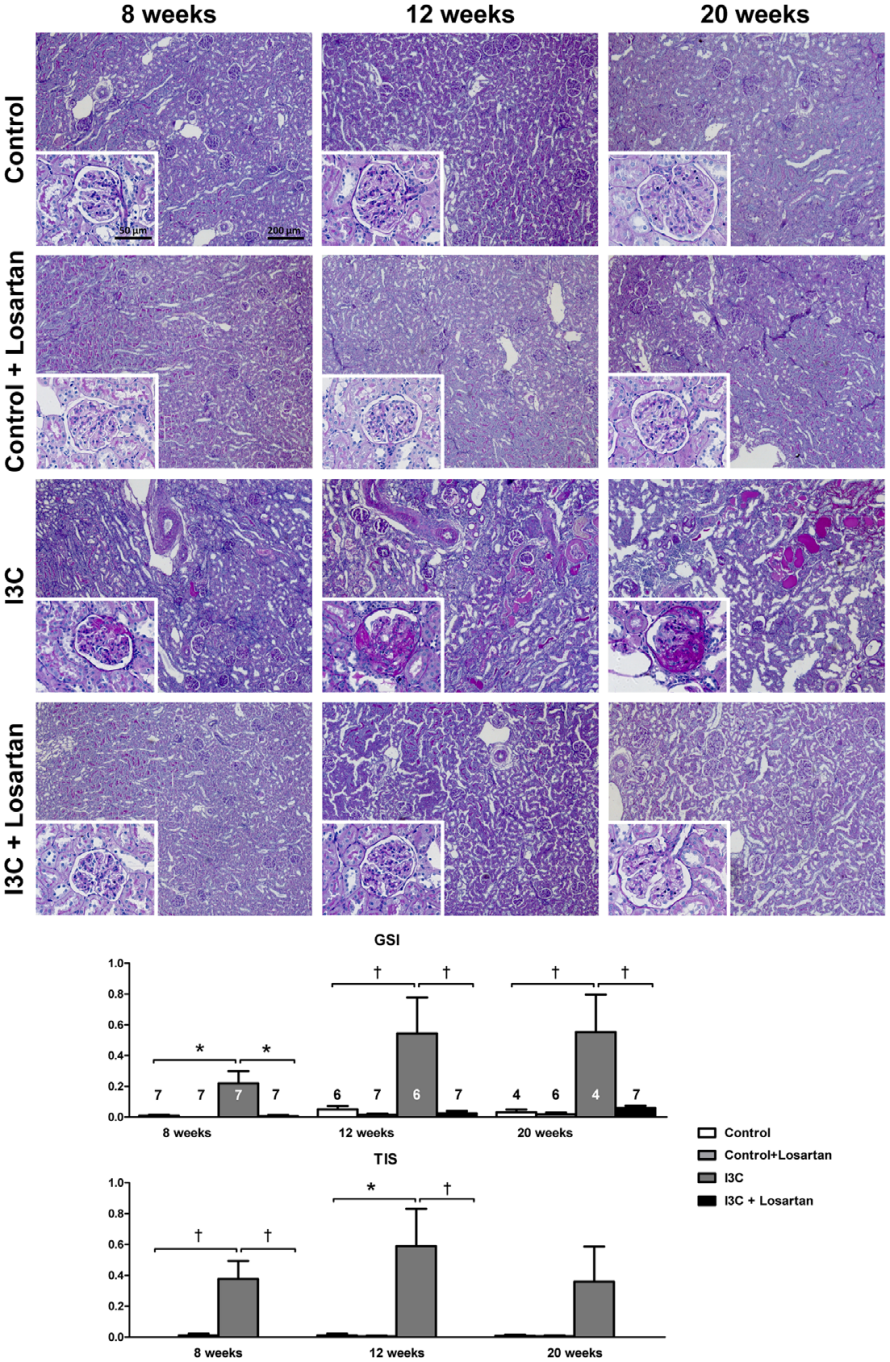
Long-term effects of AT_1-_receptor blockade on renal injury. Four weeks of RAS-stimulation induced irreversible renal damage. Despite high levels of circulating RAS-components, supplementary AT_1_R-blockade was able to completely prevent the development of renal pathology over time. The n for each group is presented in the bars of the GSI. *GSI, glomerulosclerosis index; I3C, Indole-3-Carbinol; TIS, tubulointerstitial score.*

In figure 9 the urinary excretion levels of KIM-1 and OPN are presented and demonstrate similar patterns as observed for renal gene expression. In addition, albuminuria remained significantly present even 12 weeks after stopping RAS-stimulation (Fig. 9A). Losartan fully blocked all these effects, which is again supported by the absence of renal pathology when compared with untreated age-matched RAS-stimulated rats (Fig. 10).

#### T-cell response

**Figure 11:**
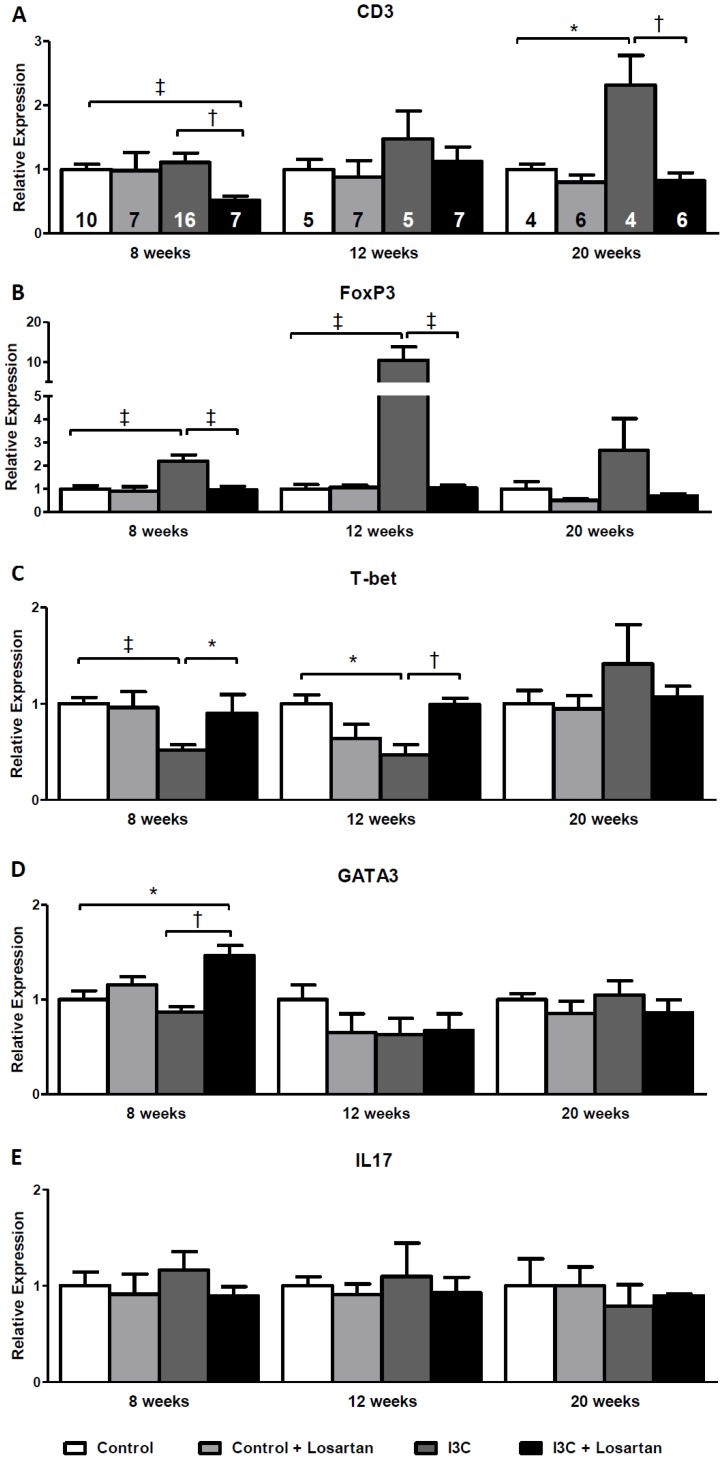
Long-term effects of AT_1_-receptor blockade on renal gene expression of T-cell markers. CD3 expression increases gradually and reaches statistical significance at 20 weeks of age in the RAS-stimulated rats. (A). FoxP3 expression demonstrated an elevation at 8 weeks of age in the RAS-stimulated rats, this increase became even more pronounced 4 weeks later and was still present, yet not significant, at 20 weeks of age (B). RAS-stimulated rats demonstrated a significant decrease in T-bet expression. This decrease was still present 4 weeks later, but disappeared at 20 weeks of age (C). Losartan treatment completely prevented the effects on CD3, FoxP3 and T-bet expression. GATA3, was only increased in the RAS-stimulated rats which received losartan treatment. After stopping stimulation and treatment GATA3 expression returned back to normal (D). Finally, no differences were observed in renal IL17 expression (E). The n for each group is presented in the bars of figure A. *FoxP3, forkhead box P3; GATA3, GATA binding protein 3; I3C, indole-3-carbinol; IL, interleukin; T-bet, T-box expressed on T-cells; Wks, weeks.*

**Figure 12:**
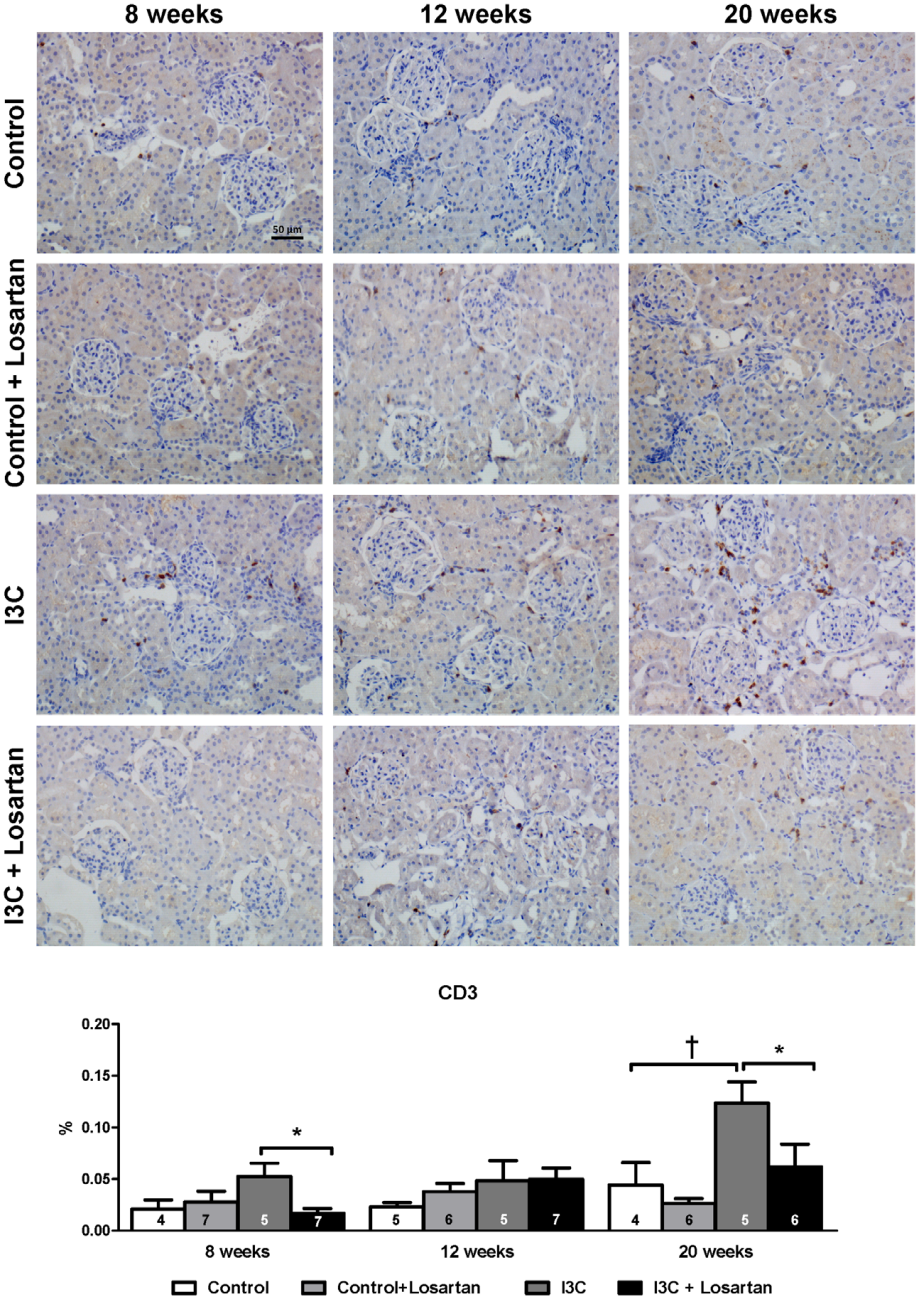
T-cell infiltration as determined by CD3 immunohistochemistry. CD3 staining of kidneys revealed a time-dependent increase of T-cells after stopping the 4 weeks of RAS-stimulation in I3C-treated animals. Losartan completely prevented this rise in T-cells. These results are in line with the data on renal gene expression. The n for each group is presented in the bars of the GSI. *GSI, glomerulosclerosis index; I3C, Indole-3-Carbinol; TIS, tubulointerstitial score.*

**Figure 13:**
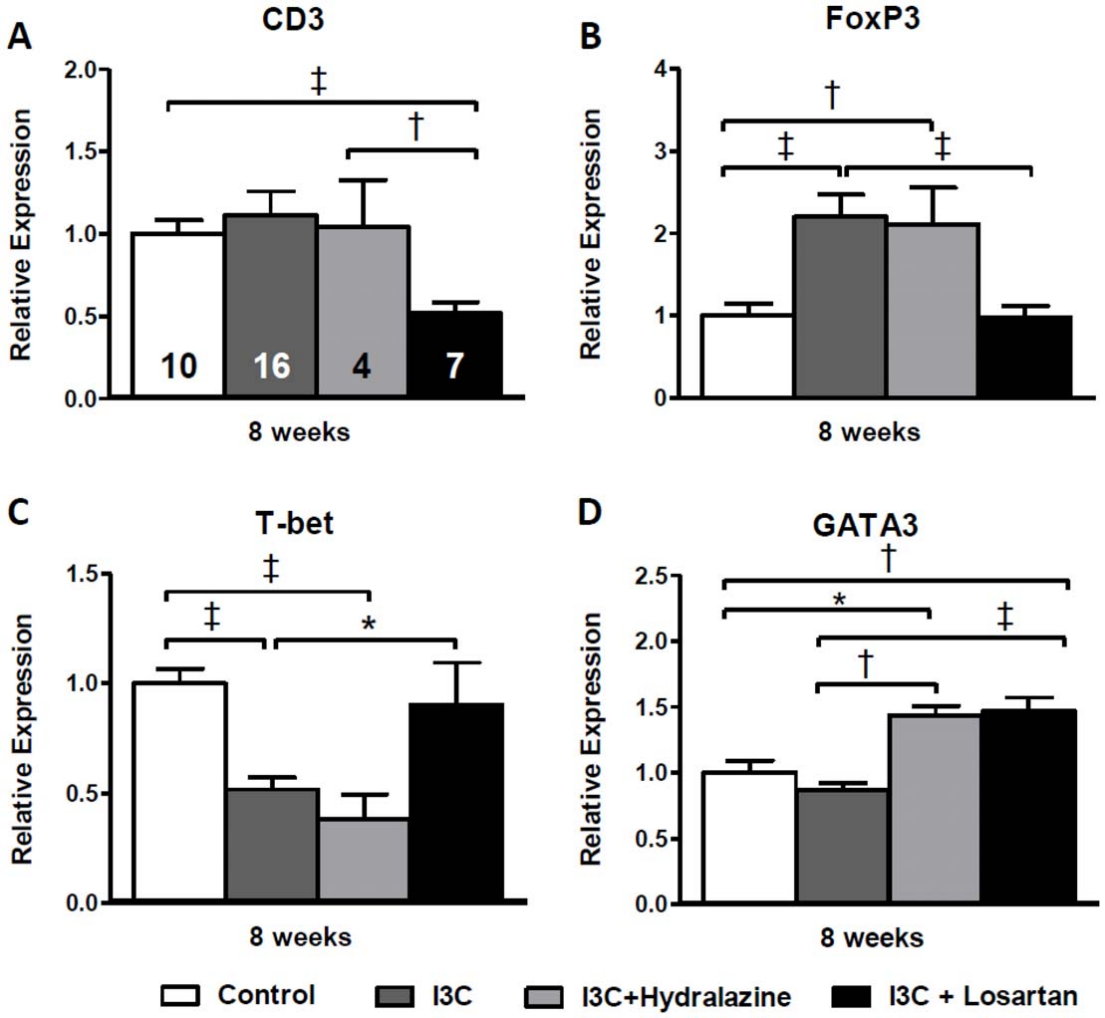
Effects of anti-hypertensive treatments on renal gene expression of T-cell markers. CD3, FoxP3 and T-bet expression were unaffected by hydralazine treatment (A-C) when compared with untreated RAS-stimulated rats. As also seen for losartan, hydralazine increased GATA3 expression (D). The n for each group is presented in the bars of figure A. *FoxP3, forkhead box P3; GATA3, GATA binding protein 3; I3C, indole-3-carbinol; T-bet, T-box expressed on T-cells; Wks, weeks.*

Renal gene expression of the general T-cell marker CD3 increased gradually and reached statistical significance at 20 weeks of age in the RAS-stimulated rats. At 8 weeks of age losartan-treatment in this group caused a decrease in CD3 expression and completely prevented the rise over time (Fig. 11A). This same pattern was confirmed by immunohistochemical staining (Fig 12). Additionally, Forkhead box protein 3 (FoxP3) expression, a marker of Tregs, was significantly elevated at 8 and 12 weeks but not anymore at 20 weeks of age. Treg function can be addressed by analysis of transforming growth factor β (TGFβ) expression. In RAS-stimulated rats the increase in Tregs did not result in an increased TGFβ expression (8 weeks: 1.13±0.08 versus 1.00±0.03 in the control group; and 12 weeks: 1.00±0.05 versus 1.00±0.08). Besides, RAS-stimulated rats demonstrated a significant decrease in T-box expressed on T-cells (T-bet) expression, a marker of T helper (Th) 1-cells. This decrease was still present 4 weeks later, but disappeared at 20 weeks of age. Losartan treatment completely prevented the effects on Treg- and T-bet expression (Fig. 11B+C). Finally, a marker of Th2-cells, GATA binding protein 3 (GATA3), was only increased in the RAS-stimulated rats which received losartan treatment. After stopping stimulation and treatment GATA3 expression returned back to normal (Fig. 11D). Finally, no differences were observed in renal interleukin (IL)17 expression (Fig. 11E). Despite the decrease in BP, renal expression of CD3, FoxP3 and T-bet was completely unaffected by hydralazine treatment when compared with untreated RAS-stimulated rats (Fig 13).

## Discussion

The new findings of this study are that sustained hypertension induced in young Cyp1a1-Ren2 rats by transient RAS-stimulation is associated with (1) increased renal expression and urinary excretion of the T-cell inducers and renal injury markers KIM-1 and OPN; (2) a specific T-cell response, consisting of an upregulation of Tregs and downregulation of Th1 cells, suggesting a shift in T-cell subpopulations, is present in the kidney and may be the underlying inducer of the irreversible renal damage in this model; and (3) the induction of the immune response is AT_1_R-mediated and might be to some extent even blood pressure independent.

### Renal Injury and Inflammation

Transient activation of the RAS in the transgenic Cyp1a1-Ren2 rat induces sustained high blood pressure and renal remodeling and damage [19], [25]. We hypothesized that the renal phenotype might be caused by a persistent inflammatory response. We therefore evaluated gene expression levels of renal glomerular and tubular injury markers. Recently, KIM-1, OPN and NGAL have been proposed as predictive biomarkers of chronic kidney disease [24]. Gene expression of all three markers was highly elevated in the kidneys of the RAS-stimulated rats and remained increased. Whereas KIM-1 in the kidney is mainly expressed in injured tubules [17], OPN is expressed in different structures of the kidney. NGAL is expressed by renal epithelial cells, but also abundantly by immune cells like neutrophils and macrophages [26]. KIM-1 and OPN are also highly expressed in several cell-types of the immune system and play a crucial role in the regulation of local inflammation. KIM-1 is expressed on both Th1 and Th2 cells and is involved in the regulation or promotion of T-cell activation [27]. OPN has pro- and anti-inflammatory effects and facilitates macrophage and T-cell recruitment to damaged tissue [28]. The elevated expression of these markers is consistent with the observation that the kidneys of RAS-stimulated rats suffer extensive damage accompanied by a local inflammatory response. The observation that KIM-1 and OPN protein excretion was highly elevated in the urine is strongly indicative for a loss of tubular function in the kidneys of these animals. This finding is enforced by the increased proteinuria/albuminuria observed in this model [19], [29]. PAI-1, a protease inhibitor closely related to the progression of glomerulosclerosis [30], increased over time in the RAS-stimulated rats. Possibly, PAI-1 is also involved in maintaining renal damage by attracting immune cells [31]. Studies in Cyp1a1-Ren2 rats indeed show increased influx of macrophages [19], [32]. Renal gene expression of nephrin was almost unaltered indicating that the glomerular filtration barrier was nearly unaffected in these rats. This finding was supported by the fact that the amount of WT-1 positive cells remained the same in the glomeruli of all groups. WT-1 is expressed by glomerular podocytes and when impaired it can result in malfunctioning of the filtration barrier via a decreased transcription of nephrin [33]. Despite an unaffected barrier the RAS-stimulated rats demonstrated high albuminuria. Cyp1a1-Ren2 rats may have a complete pressure depend albuminuria without directly affecting the barrier itself. It is known from literature that despite the elevated BP and albuminuria glomerular filtration rate is unaffected in these rats [25], [34].

AT_1_R-blockade has proven to be very effective against the BP increase in Cyp1a1-Ren2 rats [29], [35], [36]. This was also seen in the present study where BP and RVR completely returned to control values despite an elevation in plasma (pro-)renin and AngII-levels [35], [36]. More importantly, after transient RAS stimulation the losartan-treated rats remained normotensive, suggesting that no significant structural changes and/or damage occurred that would induce a hypertensive phenotype later in life. Hence, the importance of (pro-)renin receptor-((P)RR) and/or AT_2_R-activation in contributing to the renal phenotype is probably neglectable. Additionally, AT_1_R-blockade completely prevented the expression and excretion of renal injury markers and subsequently no renal damage was observed. Whereas RAS-independent BP lowering by hydralazine was able to reduce the elevation of BP in RAS-stimulated rats, KIM-1, OPN, albuminuria and renal pathology were evidently present and almost unaffected. The inability of hydralazine to decrease renal injury was also seen in a study by Shao et al. where Ang II stimulated animals showed a similar renal injury score as observed in the hydralazine-treated animals with Ang II [11]. This is a strong indication that AT_1_R-activation is the main contributor to the irreversible renal injury we observed in young Cyp1a1-Ren2 transgenic rats after transient RAS-stimulation [19]. Yet, the underlying mechanisms of this are insufficiently understood and are still subjected to intense research. Upregulation of local RAS components may be of high importance in the pathology of renal damage in these rats, an effect that can be blocked by addition of an ARB [37].

### Presence of an Underlying T-cell Mediated Immune Response

As mentioned before, the upregulation of renal KIM-1 and OPN expression could be explained by the influx of immune cells, such as macrophages and lymphocytes, in the kidney. AT_1_R-activation by Ang II is an established key factor in the initiation of the immune response and causes an upregulation of several transcription factors, chemokines and cytokines, such as NFκB, TNFα and ICAM-1. On their turn, these factors attract infiltrating immune cells and enhance the immune response, which then increases the local expression again of KIM-1, OPN and NGAL [7], [9]. Additionally, part of the Ang II effects on immunity is caused by direct action on adaptive immune cells [7]. T-cells express AT_1_R and may play a role in regulating blood pressure and target organ damage [15]. Zhang et al. demonstrate that AT_1_R on T-cells limit hypertensive injury to end-organs. Hence, a depressed T cell function is a characteristic of an adaptive immune response underlying hypertension. This concept has been proposed before by studies that put immune perturbation forward among which a depressed T cell function was suggested [38],[39]. An imbalance of T-cell subpopulations might be the root to pathological renal damage [11]. The entire T-cell population consists of a variety of subpopulations including Th1, 2 and 17 cells and Tregs, all with different functions and activation pathways. Injury resolution is an established function of Tregs. These cells typically express the transcription factor FoxP3 and normally participate in the amelioration of inflammatory-induced injury [40]. Recent studies demonstrate increases of Tregs in various organs, such as the heart and vessels, in a variety of hypertensive models. They all show that these cells play an important role in the reduction of high blood pressure and end-organ damage [1], [12], [13], [41], [42]. Th17 cells can play a role in immunity against extracellular bacteria and fungi, as well as in autoimmune reactions and diseases [43]. It was reported that IL17 produced by Th17 cells plays a critical role in maintenance of AngII-induced hypertension [44]. This study however demonstrates no difference in renal IL-17 expression, which may exclude the involvement of Th17 cells in the renal pathology observed in our model.

Nevertheless, T-cells might still be the causative factor in the irreversible renal damage observed in the Cyp1a1-Ren2 model. Renal CD3 expression was unaltered upon RAS-stimulation, however the expression increased over time indicating that there is a persistent influx of T-cells. Furthermore, the expression patterns indicate that Tregs are highly present whereas Th1 cells seem to be repressed. A possible explanation might be that there is a shift from Th1 cells to Tregs as a response to the hypertension stimulus. The Tregs are probably attracted to protect and ameliorate renal pathology by mechanisms reviewed by Bromly et al [45]. Unfortunately, they are unable to prevent the development of irreversible renal damage which might indicate a Treg dysfunction [46]. The absence of differences in TGFβ expression, a key regulator of the signaling pathways that initiate and maintain Foxp3 expression, supports this idea [47]. Whatever the mechanism, losartan-treatment completely prevented the alterations in expression of T-cell subpopulations, whereas hydralazine was unable to do so. It has been shown before that AT_1_R blockade restores T-cell population subsets back to normal [11]. This was not the result of blood pressure lowering, since hydralazine did not induce changes to the Th subsets [11]. This is in contrast to a study in which hydralazine was able to impair T-cell activation in Ang II stimulated mice [48]. It is difficult to pinpoint the cause-effect relationship between hypertension and inflammation, but the importance of this interplay and the involvement of the AT_1_R is unquestionably apparent.

In the past Lever and co-workers have intensively studied the role of low-pressor dose infusion of Ang II and its possibility to induce hypertension later in life [6], [49]. Over the years the involvement of vascular hypertrophy has been demonstrated, however, a conclusive mechanism has not been found for this interesting phenomenon. This study may provide an explanation by demonstrating the importance of AT_1_R activation in the induction of an underlying immune response. AT_1_R activation occurs already with low levels of circulating Ang II, inducing inflammatory reactions which could slowly result in remodeling processes that ultimately lead to hypertension. Of course more research would be needed to strengthen this hypothesis.

This study comes with some limitations. First, the analysis of T-cell subsets can be done by fluorescence-activated cell sorting (FACS). In addition, the data on IL17 expression is only indirect evidence that the Th17 subpopulation does not contribute to the phenotype we observe. Providing FACS data of the different T-cell subsets, including Th17 cells, would possibly strengthen the outcome of the present study. Yet, due to technical reasons these data were not acquired during the experiments. Furthermore, we state that there might be a Treg dysfunction in our model. Unfortunately, functional studies to check whether this is actually the case have not been performed and are unavailable in this study. Studies with FACS analyses and functionality tests are subjected in our current research. Despite these limitations, we believe that a significant amount of data provides evidence that T-cell aberration plays an important role in the development of renal damage in our Cyp1a1-Ren2 rat model.

### Perspectives

The clinical significance of our experimental observations is that they underscore the potential importance of inflammatory responses in the kidney to an elevation in BP at young age. This study gives a hint that at early age the induction of renal inflammation can introduce a persistent adaptive immune response which may cause pathological changes to the kidney that eventually contribute to the development of established hypertension. As seen in the present study the immune response was mainly provoked by AT_1_R-activation which might indicate that RAS blockers are essential in the treatment of high BP. Potential biomarkers for renal injury and/or renal inflammation, such as KIM-1 and OPN, should be taken into account when considering antihypertensive treatment. The focus of current treatment regiment is based upon treating patients with blood pressures above prehypertension, whereas low circulating levels of Ang II without vasoconstrictive effects can already lead to hypertension later in life. Furthermore, a modest increase in BP already induces T cell activation [48], possibly by mechanisms recently proposed by Harrison and co-workers [4] and via direct activation of AT_1_R on T-cells [15]. It would be of great interest to investigate in prehypertensive patients to which degree early renal inflammatory mechanisms are correlated to the proposed biomarkers and how they contribute to the transition to established hypertension.
